# Mechanical stretch promotes osteogenesis in the midpalatal suture by enhancing neutrophil fatty acid oxidation through the S1PR1/STAT3 axis

**DOI:** 10.1016/j.mbm.2026.100205

**Published:** 2026-07-13

**Authors:** Ting Jiang, Tian-Hao Wan, Min-Jie Wang, Xin-Yi Tan, Zhi-Chen Ling, Feng Yang, Jun Wang, Ning-Juan Ouyang

**Affiliations:** a Department of Pediatric Dentistry, Shanghai Ninth People's Hospital, Shanghai Jiao Tong University School of Medicine, China; b Department of Oral Surgery, Shanghai Ninth People's Hospital, Shanghai Jiao Tong University School of Medicine, China; c Department of Orthodontics, Shanghai Ninth People's Hospital, Shanghai Jiao Tong University School of Medicine, China; d College of Stomatology, Shanghai Jiao Tong University, China; e National Center for Stomatology, National Clinical Research Center for Oral Diseases, China; f Shanghai Key Laboratory of Stomatology, China; g Shanghai Research Institute of Stomatology, Research Unit of Oral and Maxillofacial Regenerative Medicine, Chinese Academy of Medical Sciences, China; h Oral Bioengineering Lab, Shanghai Key Laboratory of Stomatology & Shanghai Research Institute of Stomatology, China; i National Clinical Research Center of Stomatology, Shanghai, China

**Keywords:** Midpalatal expansion, Osteogenesis, Neutrophil, Fatty acid oxidation, Stretch

## Abstract

In clinical maxillary arch constriction, selecting an optimal initial distraction force may help reduce relapse associated with insufficient suture osteogenesis. Neutrophils play a crucial role in early suture osteogenesis; however, how initial distraction force regulates their function remains unclear. Therefore, this study investigated how different magnitudes of initial distraction force modulate neutrophil activity and influence suture osteogenesis. By integrating an in vivo maxillary expansion model in 6-week-old male C57BL/6 mice with flow cytometric sorting, transcriptomic analysis, and an in vitro three-dimensional stretching model, this study systematically characterized the functional and metabolic changes of neutrophils under different mechanical conditions and further explored the underlying regulatory mechanisms. The findings demonstrate that neutrophils serve as important mediators of force-dependent osteogenesis and contribute to force-dependent differences in osteogenesis during midpalatal expansion. Different magnitudes of distraction force induced metabolic divergence in intra-sutural neutrophils, leading to differential regulation of their energy metabolism and osteogenic potential. Mechanistically, mechanical tension enhanced fibroblast-derived S1P secretion via modulation of SPHK1 activity, which activated the S1PR1/STAT3 signaling pathway in neutrophils, promoted fatty acid oxidation, and ultimately drove their pro-osteogenic function. These results clarify how initial distraction force regulates the mechanics–immunometabolism–osteogenesis axis and provide a framework in which fibroblast-derived S1P and matrix stiffness coordinate neutrophil S1PR1–STAT3–FAO signaling to promote osteogenesis.

## Graphical abstract

Initial distraction force reshapes the sutural microenvironment during MPE by stimulating fibroblasts to secrete S1P. S1P activates the S1PR1–pSTAT3 signaling axis in neutrophils, enhancing fatty acid oxidation (FAO) and promoting VEGFA release. These activated neutrophils subsequently enhance the osteogenic activity of BMSCs, facilitating new bone formation within the expanding suture. This study reveals a previously unrecognized mechanics–immunometabolism–osteogenesis regulatory axis, providing new mechanistic insight and a potential therapeutic basis for optimizing clinical distraction protocols and bone regeneration strategies.

**Figure:**
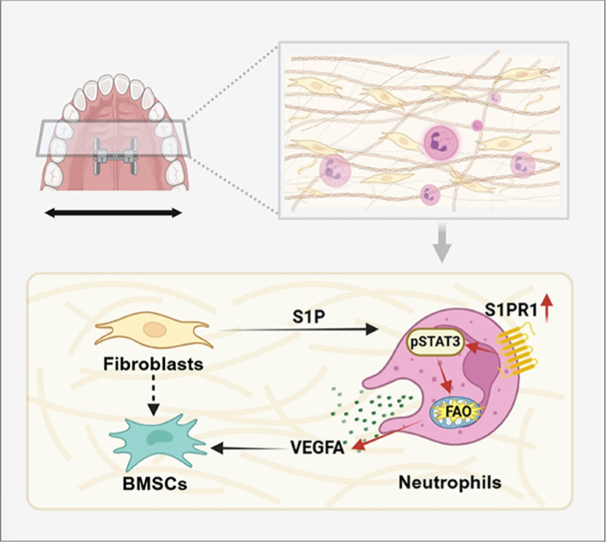

## 1 Introduction

Maxillary transverse deficiency–induced dental arch constriction has a high prevalence among Chinese adolescents, reaching 31.1%.^1^ Midpalatal expansion (MPE), as the primary treatment, essentially induces distraction osteogenesis of the midpalatal suture through mechanical force. However, current therapies often require an excessively long treatment course due to slow bone formation within the suture, and may even increase the risk of relapse.

Studies have shown that the magnitude of the initial distraction force is a key determinant of osteogenic outcomes in the suture: a force that is too small fails to effectively separate the suture, whereas an excessively large force may inhibit bone regeneration and become an important contributor to relapse.^2^ The initial expansion amount determines the magnitude of the initial distraction force applied to the suture. To effectively open the midpalatal suture and sufficiently activate sutural remodeling, orthodontists have optimized the initial activation protocol—from the traditional 0.2–0.25 mm per day to an approach of 0.4–0.5 mm for the first activation, followed by a return to 0.25 mm per day thereafter.^3^ Animal studies further indicated that setting the initial expansion to 2.5 mm (compared with 1 mm and 4 mm protocols) significantly increased new bone formation in the midpalatal suture and resulted in a more stable remodeled sutural architecture.^4^ Therefore, determining an appropriate initial distraction force is not only of significant scientific value but also a critical clinical challenge that must be addressed to guide expansion strategies.

Under distraction stimulation, a cascade of coordinated biological events promotes new bone formation within the suture, including alterations in the characteristics of sutural collagen fibers, changes in cellular behavior, and modulation of gene expression. Collagen fibers within the suture, as the principal component of the extracellular matrix, can directly sense tensile forces during distraction, leading to fiber reorientation and cellular deformation. Consequently, sutural cells and their behaviors are thought to be among the first to be influenced by the physical properties of collagen fibers.^5^ Fibroblasts are the most abundant cell type in the sutural mesenchyme; located within the fibrous matrix, they primarily maintain sutural structural stability by synthesizing type I collagen. Studies have shown that fibroblast proliferative activity is highly sensitive to the magnitude of the initial distraction force, and that both fibroblast proliferation and collagen fiber synthesis are positively correlated with increasing force intensity.^2^^,^^6^

Mesenchymal stem cells (MSCs) are key effector cells within the suture. Evidence indicates that distraction forces promote MSC proliferation at early stages, with proliferative activity positively correlated with distraction magnitude; in later stages, distraction drives the osteogenic differentiation of sutural MSCs into osteoblasts, which regulate bone matrix mineralization by secreting osteocalcin (OCN), thereby directing sutural remodeling and continued regeneration^7^, ^8^, ^9^. However, other studies suggest that OCN activation within the suture may be inhibited by stretched collagen fibers.^10^ Our previous work demonstrated that neutrophils markedly increased the OCN ^+^ area and enhanced bone volume fraction within the suture, providing the first direct evidence that neutrophils play a pivotal regulatory role in distraction osteogenesis of the suture. Notably, neutrophil numbers increased significantly by day 3 after midpalatal suture distraction, preceding the appearance of other immune cell populations—for example, macrophages were detected in the suture only after 7 days of distraction.^11^ These findings raise the question of whether the magnitude of the initial distraction force influences osteogenesis by modulating neutrophils within the suture.

Neutrophils are highly plastic and can rapidly adapt to the microenvironment, thereby adjusting their functions.^12^ Fridlender et al. described a transforming growth factor-β (TGF-β)–induced subset of tumor-associated neutrophils that, in contrast to the conventional pro-inflammatory, anti-tumor neutrophil phenotype, exhibits pro-tumor and anti-inflammatory properties and was defined as the N2 subtype.^13^ In recent years, N2 neutrophils have attracted considerable attention for their critical roles in bone regeneration through the secretion of pro-repair factors such as VEGFA and SDF1α; cytokines including IL10 have subsequently been shown to induce neutrophil polarization toward an N2 phenotype in vitro.^14^^,^^15^ Beyond cytokines, matrix stiffness also influences neutrophil functional phenotypes. Previous work established a three-dimensional neutrophil culture system using hydrogels with tunable stiffness and demonstrated that higher matrix stiffness promoted polarization toward an anti-inflammatory, pro-repair phenotype.^16^ Besides, we previously observed an increase in pro-repair neutrophils within the midpalatal suture at day 5 of distraction.^17^ These findings raise the question of whether distraction forces of different magnitudes affect osteogenesis by modulating neutrophil polarization toward the pro-repair phenotype.

Midpalatal suture distraction is a dynamic process in which mechanical stress drives continuous remodeling of the sutural matrix. Matrix remodeling can regulate mitochondrial metabolism via TGF-β signaling, thereby influencing cellular function.^18^ During distraction force–guided remodeling of the midpalatal sutural matrix, whether changes in neutrophil function are grounded in metabolic reprogramming warrants further investigation. From a mechanobiological perspective, this study innovatively proposes that the magnitude of distraction force can modulate neutrophil mitochondrial metabolism, thereby shaping their pro-osteogenic functions. This work aims to elucidate the core regulatory principles governing midpalatal suture distraction osteogenesis, provide a new paradigm for uncovering the “mechanics-metabolism-osteogenesis” interactive mechanism, and offer important scientific evidence to guide clinical orthopedic treatment.

## 2 Materials and methods

### 2.1 Animals & treatment

All experiments involving mice were approved by the Ethics Review Board of Shanghai Ninth People's Hospital (Shanghai, China) (approval number: SH9H-2025-A1658-1) and conducted in accordance with relevant ethical regulations for animal research. All procedures complied with the Animal Research: Reporting of In Vivo Experiments (ARRIVE) guidelines.

Male C57BL/6 mice, aged six weeks, were procured from the Shanghai Jihui Laboratory Animal Care (Shanghai, China). The mice were provided with powdered food and water, and the ambient temperature of the facility was maintained at 22 ± 1°C. The model for MPE in mice was established as previously described.^17^ Specifically, an opening loop constructed from stainless steel orthodontic wire (0.014-inch diameter) (GAC International Inc., Bohemia, NY, USA) was affixed with a light-cured adhesive (3M Unitek, Monrovia, CA, USA) to the first and second maxillary molars on both sides, applying initial forces of 0.40 N, 0.60 N, 0.80 N, and 1.0 N, respectively. The selected force range was designed to encompass commonly used loading levels in experimental models of midpalatal suture expansion, with approximately 0.56 N reported as an effective force for inducing sutural remodeling without excessive tissue damage^19^). Mice that did not undergo the surgical procedure served as non-expansion controls.

In the neutrophil-depleted cohort, an intraperitoneal administration of rat-anti-mouse Ly6G antibody (clone 1A8; Bio X Cell) was performed at a dosage of 200 μg per mouse two days prior to the expansion. Subsequently, the administration continued every other day at a dosage of 100 μg per mouse until seven days post-expansion. The injection of rat IgG at 200 μg per mouse was utilized as a control.

### 2.2 Micro-CT

Mice were euthanized on Day 14 after expansion, and maxillary specimens were harvested and fixed in 4% paraformaldehyde. The maxillae were then scanned using micro-computed tomography (μCT 50 cabinet microCT scanner, SCANCO Medical AG, Bassersdorf, Zurich, Switzerland) to observe the change in the palatal suture. The intact maxillae were placed in a holder and scanned with the palate perpendicular to the image plane producing a stack of 2D images or 3D volume (scan medium: air; X-ray tube potential: 45 kVp). The scanned volume had an isotropic voxel size of 8 μm. These stacks of images were then passed through a 3D Gaussian filter (with mean = 1.2 and filter support = 1). Then, quantitative analysis of the new bone formation in the suture were performed by selecting the palatal bone regions between bilateral 1st molar teeth (from the mesial aspect to the distal aspect) to obtain the bone volume fraction (BV/TV).

### 2.3 Histological analysis

The fixed samples underwent decalcification using a 10% disodium ethylenediamine tetraacetate (EDTA) solution for a duration of four weeks. Subsequently, the specimens were subjected to dehydration through a graded series of ethanol solutions. Following this, a clearing process with xylene was conducted, and the specimens were embedded in paraffin to produce paraffin sections with a thickness of 4 μm. Hematoxylin and eosin (H&E) staining was employed to evaluate osteogenesis within the midpalatal suture region. To ensure consistency throughout the study, the midpalatal suture area in the first molar tooth region was selected for analysis.

### 2.4 Fluorescence-activated cell sorting and cell culture

Mice were sacrificed on Day 1, 3, 5, or 7 after MPE under different magnitudes of mechanical stretch. The number of mice was sufficient to ensure at least five mice per group (for each time point and each mechanical stretch level). Each group was tested as an independent repeat and this experiment was repeated 3 times. The cranial–maxillary complex of each sample was split through the midpalatal suture by a blade, and tissue cells on bilateral bone surfaces inside the midpalatal suture were scraped off and added to phosphate-buffered saline (PBS) containing 10% fetal bovine serum at 4°C. The tissues were cut into pieces and gently ground. The suspension was filtered through a 400 μm filter (BD Falcon) to obtain single-cell suspensions. Red blood cells underwent lysis in ammonium chloride potassium buffer. Then, the cells were washed and resuspended in PBS, blocked with anti-FcRII/III antibody for 30 min, and stained with FITC-conjugated anti-Ly6G (1A8, BD Biosciences) and APC-conjugated anti-CD45 (#157605, BioLegend) antibodies according to the manufacturer's protocol. Immunostained Ly6G^+^CD45^+^ cells (defined as neutrophils) were sorted using a flow cytometer (Beckman Navios, USA) and and FLOWJO software (Tree Star, San Carlos, CA, USA).

Neutrophils were isolated from the bone marrow of C57BL/6 mice using a negative magnetic bead selection kit (BM, Catalog. 480058, MojoSort, BioLegend) according to the manufacturer's instructions and cultured in RPMI 1640 medium (Sigma, MO, USA).

BMSCs were isolated from the bone marrow of C57BL/6 mice (Case et al., 2010). Briefly, bone marrow was flushed out and passed through a 70 μm filter (BD Falcon, San Jose, CA). Isolated cells were resuspended in α- MEM with 10% fetal bovine serum and 1% penicillin–streptomycin (HyClone, Thermo Scientific, Logan, USA) and utilized from passages 1 to 3 in this study. The cells were cultured in humidified air with 5% CO2 at 37°C.

### 2.5 RNA-sequencing and data analysis

Flow-sorted neutrophils *a*nd fibroblasts were collected for RNA extraction (from the same batch as that used for RT–qPCR), respectively. Libraries were prepared from total RNA, and mRNA was enriched by poly(A) selection to remove rRNA. Sequencing was conducted on an Illumina NovaSeq 6000 with 2 × 150 bp reads. Trimmomatic (v0.39) cleaned the raw reads, which were then quality-checked with FastQC (v0.11.9). HISAT2 (v2.2.1) aligned the clean reads to the mouse genome (GRCm39), and StringTie (v2.1.7) estimated transcript abundance. Differential expression was analyzed in R using DESeq2 (v1.32.0) with thresholds of adjusted P < 0.05, FDR <0.1, and fold-change ≥2. Volcano plots, heatmaps, and GSEA were also generated in R.

### 2.6 FAO and ATP level analysis

FAO enzyme activities were quantitatively assessed using FAO detection reagent (#FDV-0033, Funakoshi, Japan). Cells were incubated with HBS containing the dissolved FAO Blue at 37°C for 30 min to allow for adequate staining. To assess FAO activity, the cells were visualized under a fluorescence microscope (BX53, OLYMPUS, Japan).

The fluorescent probe ATP-Red 1 (MedChem Express, NJ, USA) was used to detect mitochondrial ATP abundance as previously described.^20^ Image J software (National Institutes of Health, MD) was used to measure fluorescence intensity.

### 2.7 Fibroblast sorting and 3D coculture

Under sterile conditions, maxillae were harvested from 3-week-old C57BL/6J mice, and palatal midline suture tissues were dissected and rinsed repeatedly with PBS to remove blood and debris. The tissues were minced into 1 mm^3^ fragments and evenly placed onto the bottom of culture dishes for explant attachment; a small volume of complete medium was added to moisten the explants, and after firm adherence, additional medium was added slowly to the desired volume. Explants were cultured at 37°C with 5% CO_2_ with minimal disturbance during the first 48 h and gentle medium changes according to cell outgrowth. When cells migrated out from the explant edges and reached an appropriate confluence, explants were removed and adherent cells were dissociated and passaged for expansion, yielding a predominantly fibroblast-like adherent cell population derived from the palatal midline suture of the maxilla. After passaging and expansion, cells were incubated with APC-conjugated anti-CD45 (#157605, BioLegend) and PE-conjugated anti-Col1a1 (ab310918, Abcam), and CD45^-^Col1a1^+^ cells were sorted by flow cytometry. Purity was confirmed, with the CD45^-^Col1a1^+^ population exceeding 90%.

For 3D culture, the fibroblasts (10^5^) or/and neutrophils (10^7^) were mixed with the 1 mL GelMA solution, which were light cured with ultraviolet light (405 nm) for 10 s. Then, complete Roswell Park Memorial Institute (RPMI) 1640 medium (Sigma, MO, USA) containing 10% fetal bovine serum (FBS, Gibco, USA) was added to the culture system.

### 2.8 Application of cyclic tensile strain

The 3D coculture system was exposed to cyclic strain using an FX-5000T Flexcell system (Flexcell International Corp, NC, USA). The equiaxial cyclic stretch system was programmed to apply 5%, 10%, and 20% elongation semi-sinusoidal stretching (1.0 Hz) to the cells, respectively. Control cells were cultured under the same conditions in collagen I-coated Bioflex six-well plates without stretching (0%). The samples of the conditioned medium were added to the BMSCs medium as an inducer in the indicated experiments.

### 2.9 ALP & ARS staining and quantitation

ALP and ARS staining was performed on BMSCs with commercial kits (Beyotime, Shanghai, China) according to the manufacturer's instructions, respectively. A semiquantitative analysis of the ALP activity of BMSCs was performed by using the ALP assay kit (Beyotime, Shanghai, China). The total protein was measured using a bicinchoninic acid (BCA) assay kit (A045-4-2, Nanjing Jiancheng Bioengineering Institute, China). After ARS staining, 10% cetylpyridinium chloride was used for Alizarin red quantification and the absorbance was determined at 562 nm.

### 2.10 Real-time quantitative polymerase chain reaction (Rt-qPCR)

Total RNA was extracted using TRIzol reagent (Invitrogen, New York, NY, USA), and reverse transcription was performed with the PrimeScript RT reagent kit (Takara, Dalian, China). Rt-qPCR was performed on a LightCycler 480 II (Roche) with SYBR Green Mix (Takara, Dalian, China). The data were subjected to the comparative cycle threshold method (*ΔΔCt*) and normalized to a housekeeping gene, GAPDH. The primers for amplification are shown in Table 1.

**Table 1:** Primer used in Rt-qPCR.

Primers	Forward sequences (5′ to 3′)	Reverse sequences (5′ to 3′)
GAPDH	TCAACGGCACAGTCAAGG	ACTCCACGACA TACTCAGC
SDF1α	GCTCTGCATCAGTGACGGTA	TAATTTCGGGTCAATGCACA
VEGF	GACTGGCGAGCCTTAGTTTG	CACATCTGCAAGTACGTTCG
Il10	TTCTTTCAAACAAAGGACCAGC	GCAACCCA AGTAACCC T TAAAG
TGFβ1	GGACTCTCCACCTGCA AGAC	GACTGGCGAGCCTTAGTT TG

### 2.11 Immunofluorescence staining

After washing with phosphate buffer solution (PBS), cells underwent fixation using 4% paraformaldehyde, followed by permeabilization using a 0.1% Triton X-100 solution for 15 min. Subsequently, they were treated with 5% bovine serum albumin (BSA) for 1 h to prevent nonspecific binding. Thereafter, cells were incubated with antibodies against OCN (AF6297, Beyotime, China), S1P (#ab224618, Abcam, USA), pSTAT3-S727(#34911, CST, Boston, USA), VEGFA (#ab52917, Abcam, USA) and F-actin (TRITC-phalloidin, 40736ES75, Yeasen, China) at 4 ◦C overnight, followed by fluorescent secondary antibodies for 2 h at room temperature as previously described. Cell nuclei were identified with DAPI (C1006, Beyotime, China). Fluorescent labeling was analyzed using a fluorescent upright microscope (BX51, Olympus). Fluorescence intensities were evaluated using ImageJ software. In indicated experiments, S1P (1 μM, HY-108496, MCE) was added to the medium.

### 2.12 Mitochondrial morphology analysis

Transmission electron microscopy (TEM) was used to observe mitochondrial morphology. Cells were fixed in 4% glutaraldehyde at 4 ◦C overnight and 1% OsO_4_ at room temperature for 2 h, respectively. After dehydration with absolute alcohol, Eponate812 (Ted Pella, Redding, CA, USA) was added and cells were prepared into ultrathin slices. Subsequently, mitochondrial morphology was detected using JEM-1230 transmission electron microscope (Japan Electronics, Tokyo, Japan).

### 2.13 Western blot

Total cellular proteins of neutrophils were extracted using RIPA lysis buffer containing phenylmethanesulfonylfluoride (1:100). The extracted proteins were separated via 12% SDS-PAGE and transferred to a polyvinylidene fluoride membrane. The membrane was incubated in Tris-buffered saline with tween 20 containing 5% bovine serum albumin (BSA) for 4 h at room temperature. Afterward, the membranes were incubated with antibodies against S1PR1 (29318-1-AP, Proteintech, USA), STAT3 (#9139, CST, Boston, USA), pSTAT3-y705 (#9145, CST, Boston, USA), pSTAT3-S727(#34911, CST, Boston, USA), GAPDH (#ab8245; Abcam, Cambridge, MA, USA) at 4°C overnight, followed by peroxidase-conjugated affinipure goat anti-rabbit IgG (H + L) for 1 h at room temperature. Immunoreactivity was determined using chemiluminescence. The relative integrated density values were measured using GAPDH as a control. In indicated experiments, S1PR1 antagonists (W146, HY-101395, MCE) and STAT3 inhibitor (Stattic, HY-13818, MCE) were added to the conditioned medium.

Besides, total cellular proteins of fibroblasts co-cultured with neutrophils under various conditions were analyzed with antibodies against SPHK1 (82609-1-RR, Proteintech, USA), pSPHK1 (19561-1-AP, Proteintech, USA), S1P (#ab224618, Abcam, USA), and GAPDH (#ab8245; Abcam, Cambridge, MA, USA).

### 2.14 Atomic force microscopy (AFM)

AFM was performed with an AFM scanner (Dimension ICON, Bruker, USA) to obtain the elastic modulus of the hydrogels. For modulus testing, the experiments were performed in the force-volume mode in a fluid environment (PBS). A nanoprobe was used with a diameter of 30 nm and a spring constant of 0.05 N/m. The curves were analyzed in NanoScope Analysis software, and the Hertz model was used to calculate the elastic modulus referred to a previous study.^21^

### 2.15 Knockdown of SphK1 by siRNA transfection

Sequence-specific siRNA against SphK1 was used to silence SphK1 expression (Santa Cruz Inc., #sc-45446 & sc-365401). Fibroblasts were seeded into 6-well plates at a density of 3 × 10^4^ cells per well and transfected with siRNA (50-100 nM) using the lipofectamine RNAiMAX transfection reagent (Invitrogen, Carlsbad, CA, USA), following the manufacturer's instructions. After 48 h, SphK1 expression levels were detected by Western blot.

### 2.16 Statistical analysis

Data were presented as mean ± standard error of the Mean (SEM) from at least three biological replicates of experiments. Statistical comparison was performed using Student's t-test, one-way ANOVA with a Tukey post-hoc analysis. Significance levels are as follows: *∗P < 0.05, ∗∗P < 0.01, ∗∗∗P < 0.001, ∗∗∗∗P < 0.0001*; *ns* indicates non-significant. All statistical analyses were performed using either GraphPad Prism 9.0.

## 3 Results

### 3.1 Neutrophils mediate force-dependent differences in osteogenesis during MPE

**Fig. 1:**
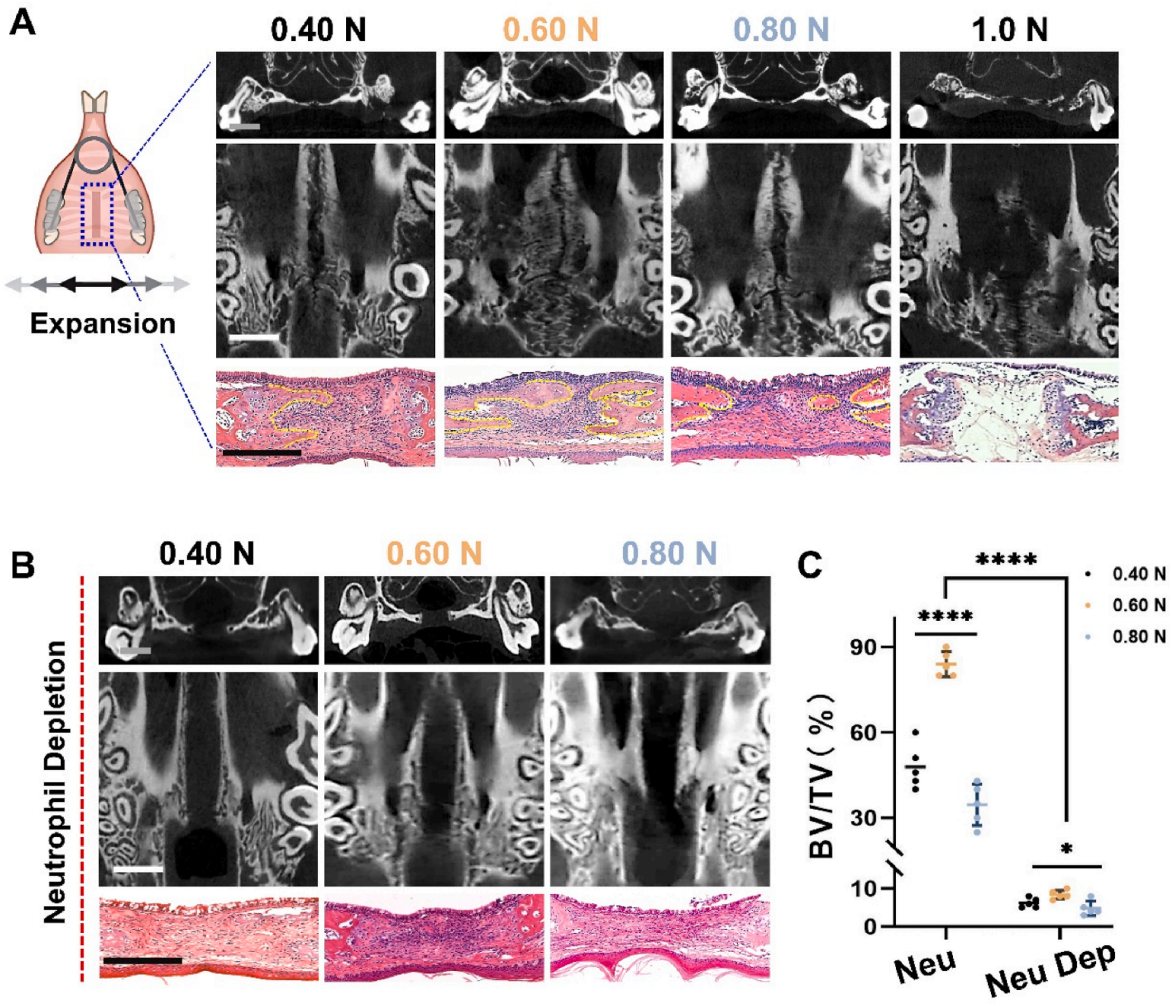
**Quantification of osteogenesis under varying force magnitudes during MPE. (A)** Suture osteogenesis induced by different distraction force magnitudes was evaluated using micro-CT and H&E staining (yellow dashed lines indicate newly formed bone). **(B)** Suture osteogenesis induced by different distraction force magnitudes after neutrophil depletion. **(C)** Quantitative analysis of the bone volume fraction (BV/TV) of intra-sutural osteogenesis. Scale bars: 1 mm for micro-CT images and 200 μm for H&E-stained sections. Data are shown as individual values with mean ± SEM (n = 5). Statistical significance is indicated as *∗P < 0.05, ∗∗∗∗P < 0.0001*.

A tooth-borne MPE model was established using different magnitudes of distraction force (0.40, 0.60, 0.80, and 1.0 N). When the distraction force was 1.0 N, midpalatal suture splitting occurred, suggesting that this load may have exceeded the physiological tolerance of the murine maxilla (Fig. 1A). Among the other three distraction force magnitudes, H&E staining revealed differences in osteogenesis (outlined by the yellow dashed line) within the expanded midpalatal suture; notably, the 0.6 N force produced the greatest amount of new bone formation.

The critical role of neutrophils in suture distraction osteogenesis has been firmly established.^17^ In subsequent experiments, circulating neutrophils were depleted via anti-Ly6G antibody injections, and the effects of different distraction force magnitudes on sutural osteogenesis were further investigated by micro-CT and H&E staining. It was found that, when the suture opening width was comparable, there were no significant differences in sutural bone formation among the different force groups, and little osteogenesis occurred overall (Fig. 1B&C).

### 3.2 Distraction force magnitude induces metabolic divergence in intra-sutural neutrophils

**Fig. 2:**
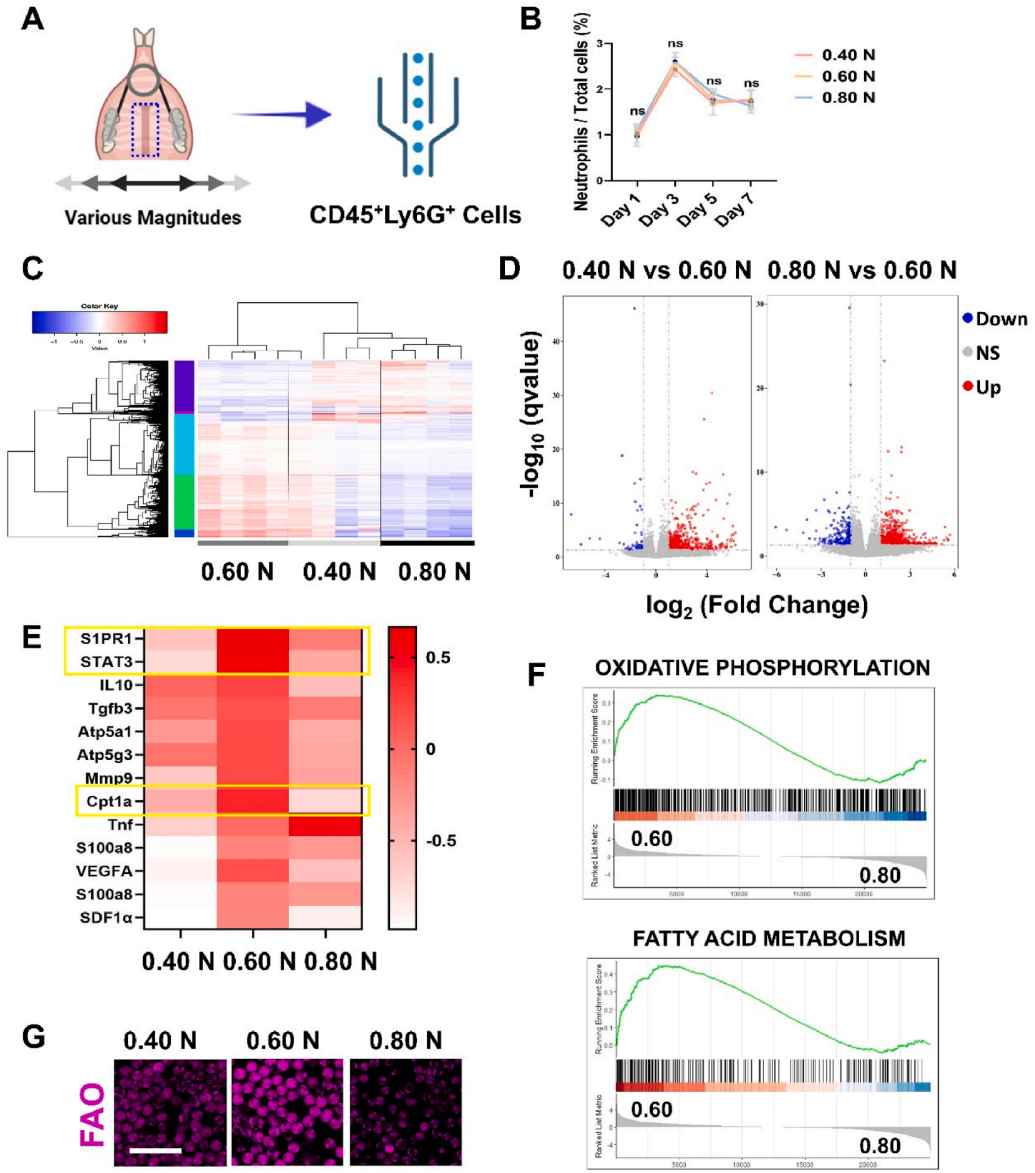
**Varying distraction force magnitudes drive metabolic divergence in sutural neutrophils. (A)** Schematic illustration of flow cytometric sorting of neutrophils (CD45^+^Ly6G^+^) within the midpalatal suture. **(B)** Quantification of neutrophils as percentages of total cells based on flow cytometry at different time points; three repeats were performed. **(C)** Heatmap visualization of RNA-seq results and cluster analysis of DEGs. Red indicates upregulated genes; blue indicates downregulated genes. **(D)** Volcano plot of the gene expression profile in neutrophils from the 0.40 N versus 0.60 N group and 0.80 N versus 0.60 N groups; up, ns, and down indicate upregulated genes, nonsignificant DEGs, and downregulated genes, respectively. **(E)** Heatmap of typical DEGs associated with neutrophil functions and metabolism. **(F)** GSEA analysis of enriched pathways associated with differentially expressed genes in sutural neutrophils under distinct tensile force magnitudes (0.60 N vs 0.80 N). **(G)** Representative images of FAO staining in sorting neutrophils. Scale bar = 50 μm. Data are shown as individual values with mean ± SEM (n = 5). Statistical significance is indicated as *ns, not significant.*

Neutrophils were isolated from the expanded midpalatal suture under different magnitudes of distraction force by flow-cytometric sorting, and their numbers were quantified (Fig. 2A). Neutrophil abundance peaked on day 3 post-expansion, whereas no significant differences in neutrophil counts were observed among force groups at the other time points (Fig. 2B). Transcriptomic profiling of the sorted neutrophils further revealed marked differences in gene-expression programs among the three groups (Fig. 2C&D). Notably, neutrophils from the 0.60 N group showed upregulated expression of S1PR1, STAT3, and carnitine palmitoyltransferase 1A (CPT1A), and increased expression of multiple pro-osteogenic genes, including IL10, SDF1α, and VEGFA (Fig. 2E). CPT1A, located on the outer mitochondrial membrane, is a key rate-limiting enzyme in the carnitine shuttle that enables long-chain fatty acids to enter mitochondria for β-oxidation (fatty acid oxidation, FAO); its upregulation generally indicates enhanced reliance on FAO for energy production. Gene set enrichment analysis (GSEA) further revealed that, compared with the 0.80 N group, differentially expressed genes in 0.6 N-neutrophils were coordinately enriched in the oxidative phosphorylation (OXPHOS) and FAO pathways, consistent with a shift toward mitochondrial respiration– and FAO-associated metabolic reprogramming rather than changes driven by single genes (Fig. 2F). This metabolic phenotype was corroborated by FAOBlue fluorescence staining (Fig. 2G).

### 3.3 Neutrophils are important mediators of force-dependent osteogenesis in vitro

**Fig. 3:**
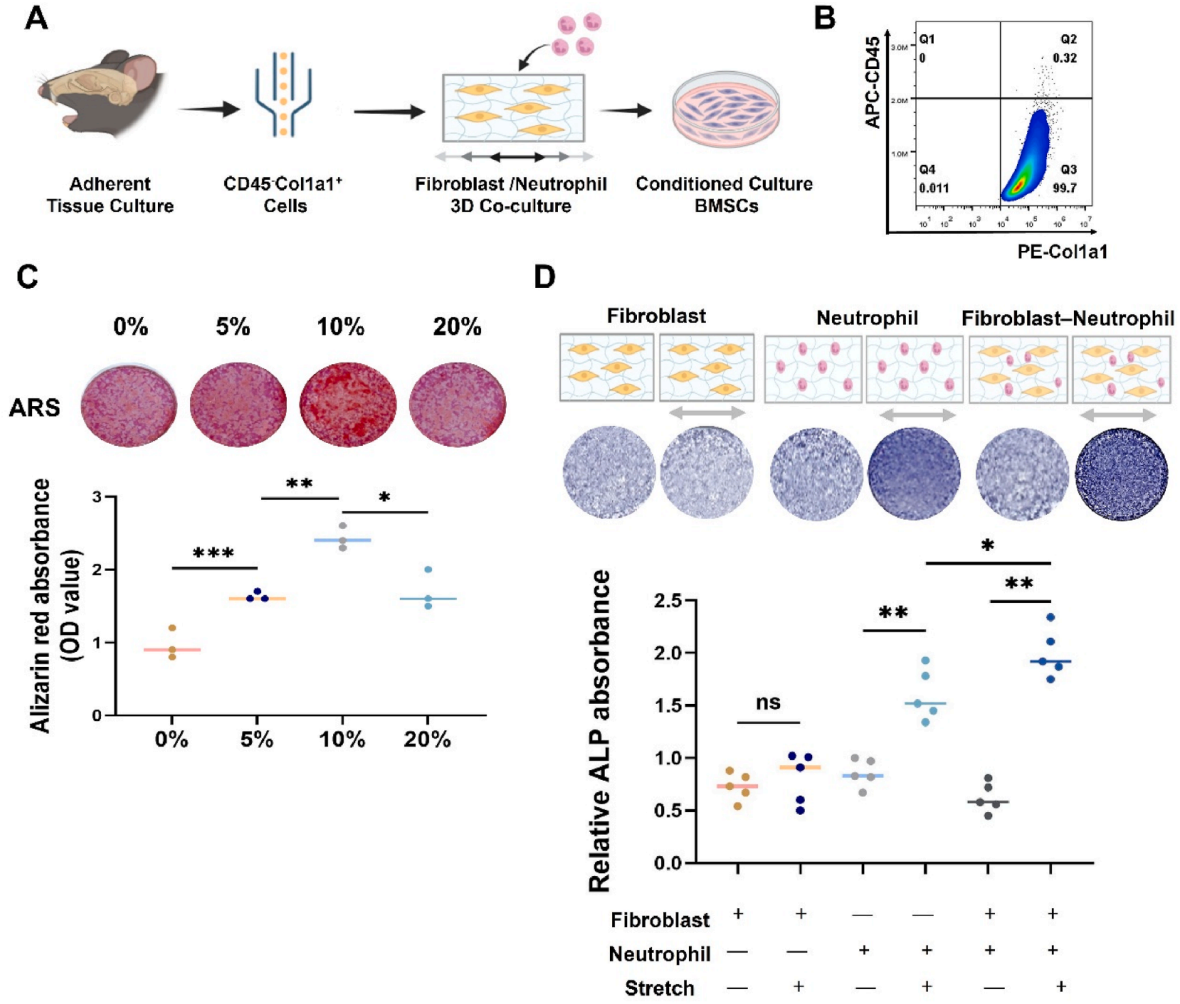
**Neutrophils play a crucial role in mediating force-dependent osteogenesis. (A)** Schematic illustration of maxillary midpalatal suture fibroblast isolation, establishment of a 3D distraction co-culture system, and BMSC conditioning with conditioned medium. **(B)** Flow cytometry confirmed that the isolated fibroblasts reached a purity of 99.7%. **(C)** ARS staining and mineralization was quantified by colorimetric analysis of the Alizarin Red S eluent from calcium nodules (n = 3). **(D)** Component-wise dissection of the 3D distraction microenvironment: ALP staining and quantitative assessment of ALP activity (n = 5). Data are shown as mean ± SEM; significance is indicated as *ns, not significant; ∗P < 0.05, ∗∗P < 0.01, ∗∗∗P < 0.001.*

Further in vitro investigations were conducted to elucidate the mechanisms underlying neutrophil functional and metabolic changes. After midpalatal suture expansion, mouse maxillary bone explants were cultured, and fibroblasts (CD45^-^Col1a1^+^) were isolated by flow sorting. A 3D in vitro distraction system with fibroblasts and/or neutrophils was established, and conditioned media were collected to assess pro-osteogenic effects on BMSC osteogenic differentiation (Fig. 3A&B). First, different distraction stretch were applied to the 3D fibroblast–neutrophil co-culture system, and conditioned media were used to culture BMSCs. ARS staining showed that 10% stretch significantly enhanced the pro-osteogenic capacity of this system (Fig. 3C). Further analysis of the system components showed that stretched fibroblasts had a limited pro-osteogenic effect, whereas stretched neutrophils markedly promoted osteogenesis; notably, neutrophils co-cultured with fibroblasts exhibited an even stronger pro-osteogenic effect (Fig. 3D).

### 3.4 Distinct distraction forces differentially modulate neutrophil energy metabolism and pro-osteogenic capacity

**Fig. 4:**
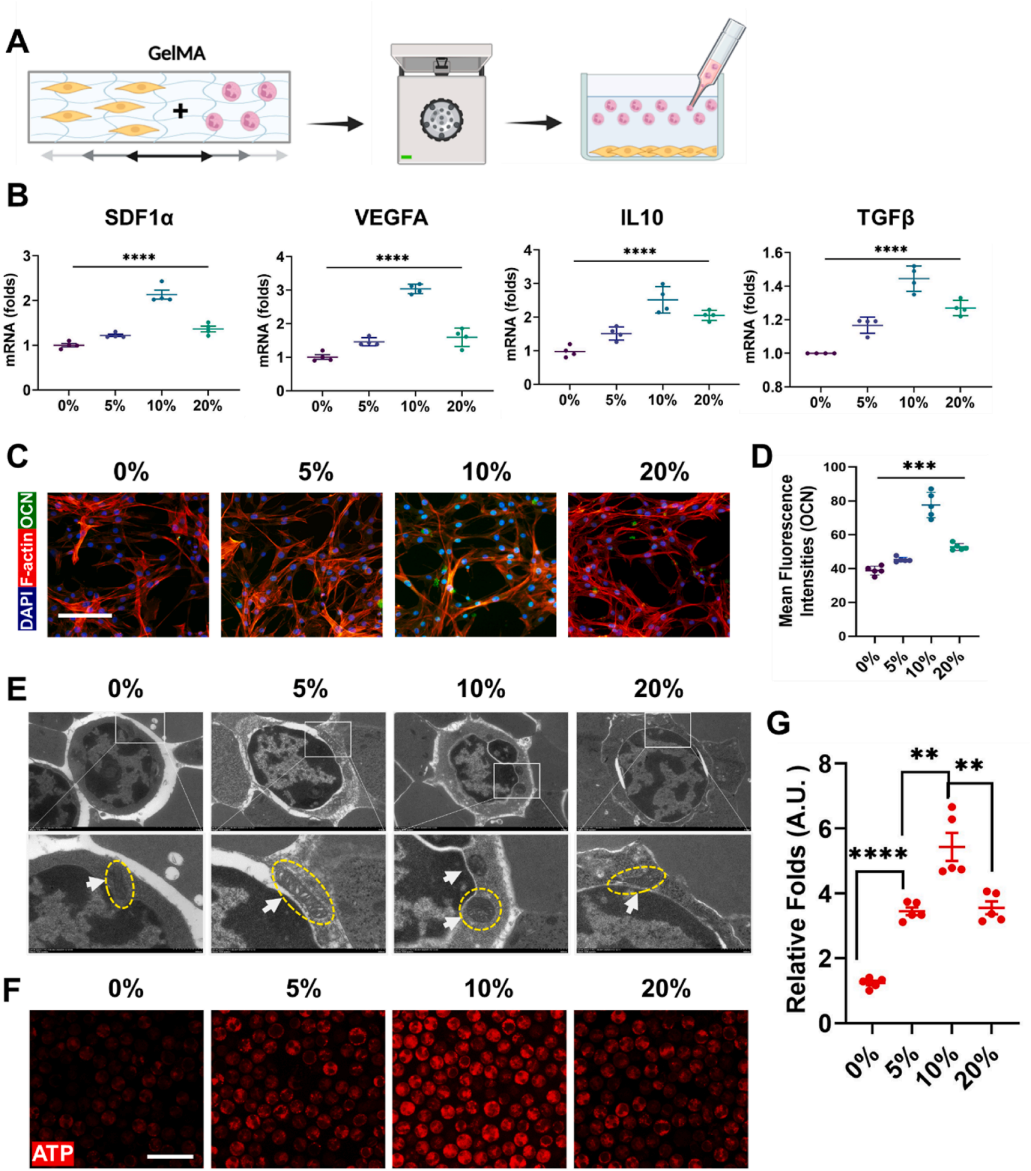
**Distinct distraction force magnitudes modulate neutrophil energy metabolism and pro-osteogenic activity. (A)** Schematic diagram of neutrophil isolation from the co-culture system by differential centrifugation. **(B)** RT–qPCR analysis of pro-osteogenic gene expression in neutrophils under different distraction microenvironments. Data are presented as fold change (n = 4). **(C&D)** Immunofluorescence staining showing OCN expression intensity in BMSCs under different conditioned media, with quantitative analysis. Scale bar = 10 μm**. (E)** Representative TEM images of neutrophil mitochondria under different distraction microenvironments. **(F&G)** Representative fluorescence micrographs of ATP staining in neutrophils exposed to distinct distraction microenvironments, together with corresponding quantitative analyses (n = 5). Scale bar = 50 μm. Data are shown as mean ± SEM. Statistical significance: *∗∗P < 0.01, ∗∗∗P < 0.001, ∗∗∗∗P < 0.0001.*

Neutrophils were isolated from a three-dimensional co-culture system by differential centrifugation, and the effects of different distraction microenvironments on neutrophil function were assessed (Fig. 4A). Rt-qPCR analysis showed that a 10% distraction microenvironment significantly upregulated the expression of pro-osteogenic genes in neutrophils, including SDF1α, VEGFA, IL10, and TGFβ (Fig. 4B). Immunofluorescence further confirmed that conditioned medium generated under the 10% distraction condition markedly increased OCN expression in BMSCs, indicating enhanced pro-osteogenic potential (Fig. 4C&D). Transmission electron microscopy (TEM) revealed predominantly round mitochondria in neutrophils under the 10% distraction condition, whereas mitochondria were mostly elongated under other conditions, suggesting altered mitochondrial dynamics and metabolic state (Fig. 4E). Consistently, ATP staining demonstrated higher intracellular ATP levels in neutrophils exposed to the 10% distraction microenvironment, indicating a more robust energy supply (Fig. 4F&G).

### 3.5 Mechanical stretch modulates neutrophil FAO via the S1PR1/STAT3 axis to regulate pro-osteogenic function

**Fig. 5:**
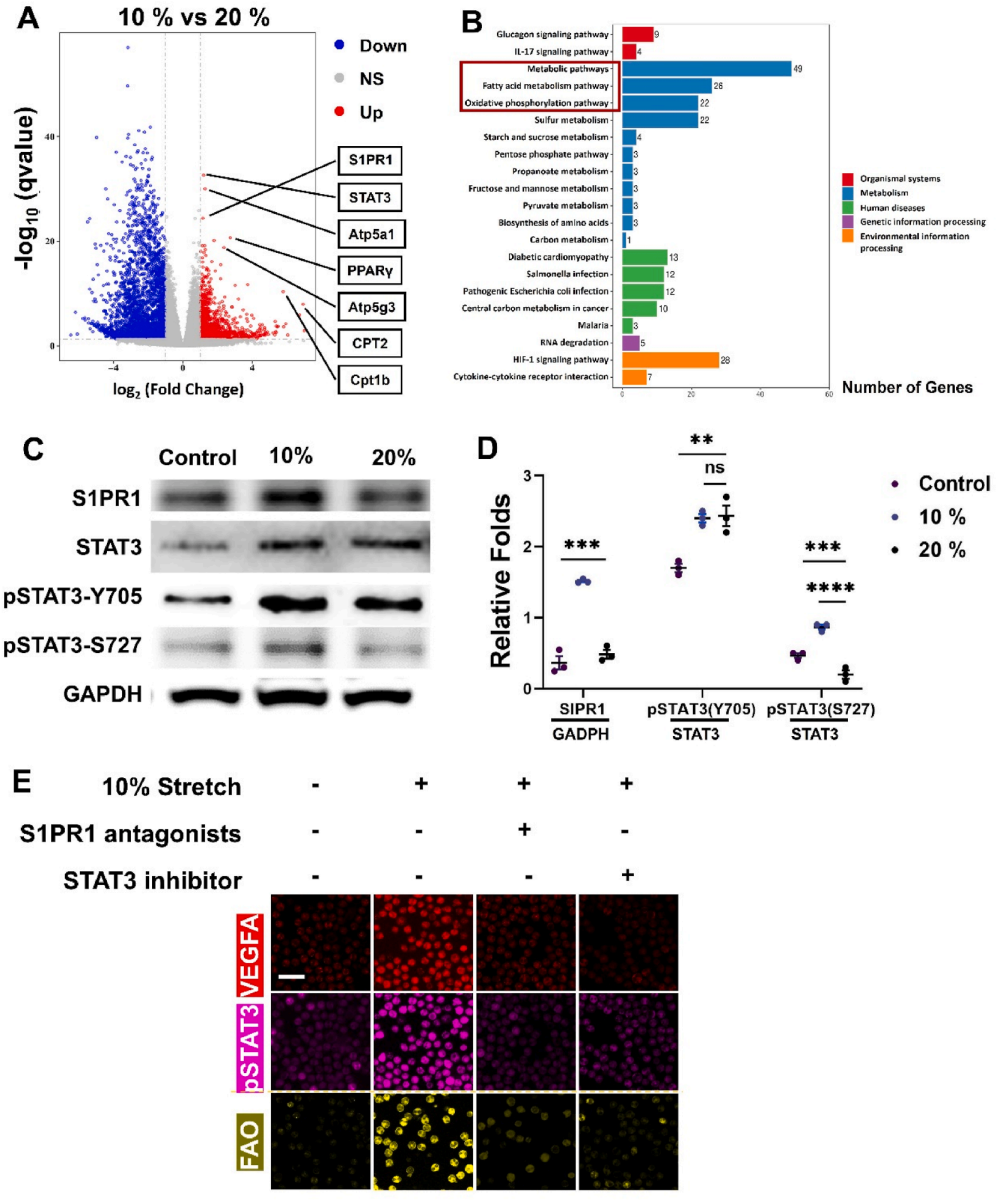
**Mechanical Stretch Tunes Neutrophil FAO Through the S1PR1–STAT3 Axis to Promote Pro-osteogenic Activity. (A)** Heatmap of representative DEGs in neutrophils under 10% and 20% distraction microenvironments. **(B)** GO enrichment analysis of DEGs in neutrophils under 10% versus 20% distraction microenvironments. **(C&D)** Representative Western blot images and quantitative analysis of S1PR1 expression and STAT3 activation in neutrophils under different distraction microenvironments (n = 3). **(E)** FAO, STAT3 phosphorylation, and VEGFA expression in neutrophils under different conditions. Data are presented as mean ± SEM; Statistical significance: *ns, not significant, ∗∗P < 0.01, ∗∗∗P < 0.001, ∗∗∗∗P < 0.0001.*

Transcriptomic profiling of neutrophils exposed to 10% versus 20% distraction microenvironments revealed a characteristic upregulation of FAO–related genes in the 10% distraction group (Fig. 5A). Consistently, Gene Ontology enrichment analysis indicated a significant enhancement of FAO and oxidative metabolic pathways in neutrophils under 10% distraction (Fig. 5B). Mechanistically, Western blotting showed increased expression of the neutrophil membrane protein S1PR1 in the 10% distraction microenvironment (Fig. 5C). In parallel, STAT3 phosphorylation at Ser727 was markedly elevated, whereas phosphorylation at Tyr705 showed no appreciable change (Fig. 5C&D). Collectively, these findings suggest that the 10% distraction microenvironment promotes STAT3 (Ser727) phosphorylation in neutrophils, thereby enhancing FAO and increasing VEGFA expression. Functional experiments further supported this pathway. Treatment with an S1PR1 antagonist reduced STAT3 phosphorylation, accompanied by decreased FAO and lower VEGFA levels (Fig. 5E). Similarly, inhibition of STAT3 phosphorylation led to reduced STAT3 phosphorylation, downregulated FAO, and decreased VEGFA expression.

Together, these results indicate that the S1PR1–STAT3 (Ser727) axis may be a key signaling pathway through which the 10% distraction microenvironment drives metabolic reprogramming (enhanced FAO) in neutrophils and promotes VEGFA expression.

### 3.6 Mechanical Tension Regulates Fibroblast S1P secretion by modulating SPHK1 enzymatic activity

**Fig. 6:**
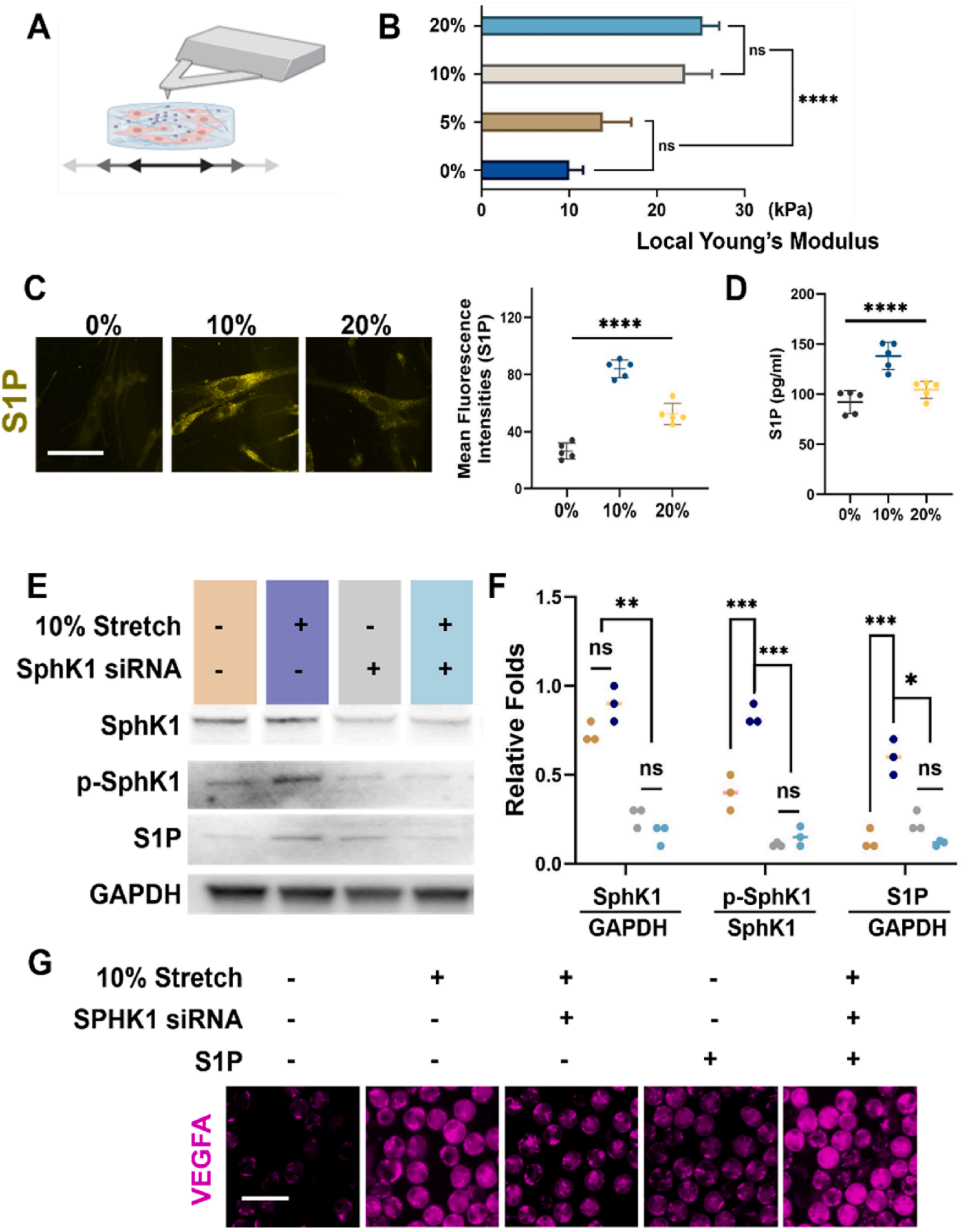
**Tension Regulates Fibroblast S1P Secretion via SPHK1. (A&B)** Schematic illustration and quantitative analysis of matrix stiffness in the 3D distraction (stretch) system measured by AFM. **(C)** Representative immunofluorescence images of S1P in fibroblasts and quantitative analysis (n = 5). Scale bar = 10 μm. **(D)** The concentration of S1P was measured by ELISA. **(E&F)** Western blot showing SPHK1 expression and S1P activation in fibroblasts under different conditions, with quantitative analysis (n = 3). **(G)** Representative immunofluorescence images of VEGFA in neutrophils under different conditions. Scale bar = 20 μm. Data are presented as mean ± SEM; Statistical significance: *ns, not significant, ∗P < 0.05, ∗∗P < 0.01, ∗∗∗P < 0.001, ∗∗∗∗P < 0.0001.*

To elucidate the upstream mechanisms driving neutrophil S1PR1 activation within the distraction microenvironment, a series of follow-up experiments was conducted. First, matrix mechanics were assessed by AFM (Fig. 6A). Both 10% and 20% distraction markedly increased the stiffness of the 3D culture matrix, with no significant difference between the two stretch magnitudes (Fig. 6B). We then evaluated alterations in sphingosine-1-phosphate (S1P), the cognate ligand of S1PR1, in the 10% and 20% distraction systems. Immunofluorescence staining of isolated fibroblasts revealed a pronounced upregulation of S1P in fibroblasts exposed to the 10% distraction microenvironment (Fig. 6C). Besides, the level of S1P in the conditioned medium was significantly upregulated (Fig. 6D).

Given that sphingosine kinase 1 (SPHK1) is a key enzyme for S1P biosynthesis, we next interrogated its role mechanistically. siRNA-mediated silencing of sphk1 in fibroblasts significantly reduced SPHK1 expression and diminished S1P production. Notably, even under distraction conditions, sphk1 knockdown continued to suppress downstream S1P levels (Fig. 6E&F). Collectively, these data indicate that mechanical stretch modulates fibroblast-derived S1P production in a SPHK1-dependent manner within the distraction microenvironment.

At the neutrophil level, reduced S1P availability from sphk1-silenced fibroblasts was accompanied by a concomitant decrease in VEGFA expression. In the absence of distraction, exogenous S1P supplementation elicited only a limited increase in neutrophil VEGFA, suggesting that neutrophil responses may also be constrained by matrix mechanical cues such as stiffness. By contrast, in the distraction microenvironment, S1P supplementation robustly rescued neutrophil VEGFA expression (Fig. 6G).

## 4 Discussion

Maxillary expansion induces new bone formation by applying intermittent, short-distance mechanical distraction to the suture, thereby promoting the deposition of bone mineralized matrix between the separated bone edges. Bone-anchored expansion techniques overcome the histological limitation of midpalatal suture ossification, enabling successful suture opening in late adolescents and adult patients. However, this approach still faces challenges of limited efficacy and unstable outcomes. Clinical studies have shown that approximately 50% of the gained midpalatal suture width is lost on average one year after expansion treatment.^22^

This study demonstrated that different magnitudes of distraction force result in marked differences in early osteogenesis within the midpalatal suture. In the present study, different mechanical loading conditions were applied in vivo and in vitro to investigate biological responses under graded mechanical stimulation. The in vivo distraction forces (0.40-1.0 N) represent commonly used loading levels in murine midpalatal expansion models. In contrast, the in vitro cyclic stretch system applies strain (% deformation) rather than force, reflecting a different mechanical parameter at the cellular level. Importantly, the in vitro stretch magnitudes (5%, 10%, and 20%) were not intended to directly correspond to specific in vivo force values. Instead, they were designed to simulate graded mechanical stimuli and to explore the dose-dependent cellular responses to increasing mechanical loading. This approach is widely used in mechanobiology studies, where strain-based in vitro systems are employed to model the biological effects of force-induced mechanical environments in vivo. When neutrophils were depleted, the expansion width of the maxillary midpalatal suture remained comparable between groups, yet osteogenesis was largely absent and no significant differences in bone formation were observed. These findings suggest that neutrophils are involved in sutural distraction-induced osteogenesis, and that the differential osteogenic outcomes induced by varying distraction forces are likely mediated by neutrophil activity. Further investigation revealed that varying magnitudes of distraction force did not significantly alter the number of neutrophils recruited to the midpalatal suture, suggesting that neutrophils recruited during the early phase of distraction undergo qualitative, rather than quantitative, changes.

Neutrophils (Ly6G^+^CD45^+^) from the midpalatal suture under different magnitudes of distraction force were sorted and subjected to transcriptomic sequencing. The results showed that, in the experimental group exhibiting the most abundant early sutural osteogenesis, neutrophils displayed a pro-repair phenotype (N2-like). Mitochondrial metabolism–related genes, including *Atp5a1, Atp5g3, and Cpt1a*, were upregulated. GSEA further revealed significant activation of FAO and OXPHOS pathways in these neutrophils. *Cpt1a* encodes carnitine palmitoyltransferase 1A (CPT1A), the rate-limiting enzyme of FAO. CPT1A is responsible for transporting long-chain fatty acids into mitochondria for β-oxidation, thereby providing energy for cellular metabolism. Studies have shown that upregulation of *Cpt1a* expression can markedly enhance fatty acid oxidation and consequently improve cellular energy metabolic capacity.^23^ In one study, aged obese mice receiving long-term supplementation with docosahexaenoic acid and aerobic exercise exhibited significantly increased *Cpt1a* expression, which was accompanied by enhanced fatty acid oxidation and downregulation of inflammatory genes.^24^ In immune regulation, butyrate enhances FAO by upregulating CPT1A activity, thereby promoting the differentiation of induced regulatory T cells through its conversion into butyryl-coenzyme A, which counteracts the binding of malonyl-coenzyme A, highlighting the important role of CPT1A in maintaining immune homeostasis.^25^

In vitro studies also revealed that, within an appropriate distraction microenvironment, neutrophil energy metabolism is closely associated with their pro-osteogenic capacity. However, these findings do not establish a causal role for FAO in neutrophil-mediated osteogenesis, and further validation using FAO-specific inhibition or rescue strategies might be required. Studies have shown that mitochondrial FAO plays a critical role in maintaining cellular homeostasis and in cellular responses to mechanical stimuli.^26^ Changes in mitochondrial morphology are not only a manifestation of cellular adaptation to external stress but may also represent an intrinsic mechanism for metabolic regulation. Mitochondria are central regulators of cellular stress responses and metabolic homeostasis, a concept supported by numerous studies. Structural alterations in mitochondria can modulate energy metabolism by influencing the stability of OXPHOS complexes, thereby affecting cellular energy expenditure and metabolic state.^27^ In addition, mitochondria play essential roles in regulating cell death pathways, including apoptosis and autophagy, both of which are closely linked to cellular metabolic status.^28^ Mitochondrial morphological dynamics are also closely associated with cellular responses to environmental stress. Under heat stress conditions, mitochondria help protect cells from damage by regulating calcium homeostasis and oxidative stress responses.^29^ Moreover, mitochondrial dynamics are involved in immune regulation. For instance, in tumor immune regulation, mitochondria can influence cell proliferation and apoptosis through modulation of the cGMP/PKG1 signaling pathway.^30^ Importantly, mitochondrial morphological changes are not merely passive responses to external stress but may also represent an active mechanism for metabolic adaptation. In obesity-associated metabolic disorders, mitochondria regulate energy metabolism by remodeling cristae architecture, thereby influencing energy expenditure and lipid storage in adipocytes.^27^

Besides, it was found that under distraction stimulation of appropriate intensity, the expression levels of S1PR1 and phosphorylated STAT3 (pSTAT3) in neutrophils were markedly increased. S1PR1 can enhance cell survival by promoting sustained activation of STAT3, while STAT3 can in turn induce the expression of S1PR1, forming a self-reinforcing positive feedback loop known as the S1PR1–STAT3 cycle.^31^ The phosphorylation sites of STAT3 include tyrosine residue Y705 and serine residue S727. Phosphorylation at S727 drives the translocation of STAT3 to mitochondria, where it regulates the activity of mitochondrial electron transport chain complexes. This process helps maintain normal OXPHOS, thereby improving ATP production efficiency. Meanwhile, enhanced STAT3 activity can promote FAO and increase the production of acetyl-CoA, providing additional substrates for the tricarboxylic acid cycle.^32^^,^^33^ Accumulation of acetyl-CoA can further regulate the expression of FAO-related genes, thereby creating a positive feedback mechanism that enhances cellular fatty acid oxidation.^34^^,^^35^

We further investigated the molecular mechanisms underlying the metabolic and functional differences in neutrophils. We first examined changes in matrix stiffness; however, this factor alone could not explain why neutrophils fail to promote osteogenesis in a microenvironment subjected to higher distraction stimulation.^36^ S1P is the primary ligand for S1PR1, and SphK1 is the key enzyme responsible for its production. As an important signaling molecule, S1P participates in the regulation of a wide range of cellular functions. Previous studies have shown that the SphK1/S1P axis plays critical roles in cell proliferation, apoptosis, inflammatory responses, and fibrosis. First, the role of SphK1 in cell proliferation has been extensively investigated. Activation of SphK1 can promote cell proliferation through the ERK signaling pathway. In epithelial ovarian cancer cells, adipocytes enhance cell proliferation by activating SphK1 in an ERK phosphorylation–dependent manner.^37^ In addition, the involvement of SphK1 in fibrotic processes has also been demonstrated. In cardiac fibrosis, SphK1 promotes fibrogenesis through an autocrine S1P signaling mechanism.^38^ Second, the role of S1P in inflammatory responses is also well recognized. S1P can promote the release of inflammatory cytokines through activation of S1P receptors, particularly S1P1 and S1P3. In lung adenocarcinoma cells, S1P induces TNF-α release via the TLR9 signaling pathway, a process that depends on S1P production and extracellular export.^39^ Finally, the regulatory roles of SphK1 and S1P under pathological conditions have also attracted increasing attention. In ischemia–reperfusion injury, inhibition of the SphK1/S1P signaling pathway alleviates liver damage, suggesting that this pathway plays an important regulatory role in pathological contexts.^40^ Taken together, SphK1 and S1P in fibroblasts are involved in multiple physiological and pathological processes. By regulating cell proliferation, inflammatory responses, and fibrosis, the SphK1/S1P axis influences cellular function and state. These findings not only highlight the biological importance of SphK1 and S1P but also provide new insights for the treatment of related diseases. However, it should be also noted that, S1P within the tissue microenvironment may not be exclusively derived from fibroblasts. Other cell types could also contribute to its production and regulation. Although our in vitro data support fibroblasts as a major source of S1P under mechanical stimulation, the relative contributions of different cell populations in vivo remain unclear. Therefore, further studies are required to systematically delineate the cellular sources of S1P and their respective roles under mechanical conditions.

In summary, this study shows that initial mechanical traction alters the stiffness of the palatal suture matrix while simultaneously stimulating fibroblasts—the major cellular component of the suture—to secrete S1P. The released S1P subsequently activates S1PR1 on recruited neutrophils, triggering STAT3 phosphorylation at the S727 site and its translocation to mitochondria. This mitochondrial STAT3 signaling is associated with enhanced FAO, thereby augmenting osteogenic differentiation capacity and ultimately promoting early distraction-induced osteogenesis within the palatal suture. Notably, mechanobiological regulation during midpalatal suture expansion operates across interconnected timescales. Cyclic mechanical stimulation at the seconds-to-minutes timescale is rapidly sensed by resident cells and converted into biochemical signals through mechanotransduction pathways. These early events are subsequently linked to immune cell recruitment and functional adaptation over days, including the neutrophil infiltration and metabolic reprogramming observed in this study. At later stages, these processes contribute to osteogenesis and extracellular matrix remodeling, ultimately leading to tissue regeneration. Thus, mechanical signaling, immunometabolic regulation, and tissue remodeling form a continuous cascade rather than discrete events. Within this framework, our findings support a model in which neutrophils serve as an important intermediary linking early mechanical cues to downstream osteogenic outcomes. This multiscale perspective helps explain how short-term mechanical stimuli are translated into long-term structural changes during force-induced bone formation.

Nevertheless, several limitations should be noted. This study was conducted exclusively in young male mice, and thus the generalizability of the findings to females or aged individuals remains uncertain. In addition, long-term outcomes following distraction were not evaluated. Finally, given the species-specific dentoalveolar characteristics of rodents, caution is warranted when extrapolating these results to human clinical settings.

## 5 Conclusion

This study identifies initial distraction force as a key mechanical determinant of sutural osteogenic outcomes. It promotes fibroblast secretion of S1P and induces changes in matrix stiffness within the suture; together, these cues reshape the local microenvironment and regulate the neutrophil S1PR1-pSTAT3 axis. This signaling program drives a metabolic shift toward enhanced FAO, thereby strengthening neutrophil pro-osteogenic activity and ultimately influencing new bone formation and remodeling within the suture. Collectively, our findings delineate a core regulatory cascade linking distraction force, fibroblast secretory activity, matrix mechanics, neutrophil signaling and metabolism, and osteogenic function. This work provides a framework for understanding the mechanics–metabolism–osteogenesis interplay, advances integration between mechanobiology and immunometabolic regulation, and offers a rationale and potential translational avenues for optimizing clinical distraction protocols and developing targeted interventions in bone tissue engineering and regenerative medicine.

## Data availability

Data will be made available on request.

## Ethical approval

The animal experiments were conducted in accordance with the ethical guidelines approved by the Ethics Review Board at Shanghai Ninth People's Hospital (Shanghai, China) (SH9H-2025-A1658-1).

## CRediT authorship contribution statement

**Ting Jiang:** Funding acquisition, Writing – original draft, Writing – review & editing. **Tian-Hao Wan:** Investigation, Methodology, Project administration. **Min-Jie Wang:** Resources, Software, Supervision. **Xin-Yi Tan:** Funding acquisition, Methodology, Software, Visualization. **Zhi-Chen Ling:** Methodology, Supervision, Visualization. **Feng Yang:** Formal analysis, Project administration, Validation. **Jun Wang:** Funding acquisition, Validation, Visualization. **Ning-Juan Ouyang:** Conceptualization, Data curation, Formal analysis.

## Declaration of competing interest

The authors have declared no conflict of interest.
